# Features that Influence Fast Disease Progression in Behavioral Variant Frontotemporal Dementia

**DOI:** 10.1002/alz70857_104587

**Published:** 2025-12-25

**Authors:** Molly Split, Daliah Ross, Zachary J. Kunicki, Rachel M Keszycki, Sarah Prieto, Masood Manoochehri, Edward D. Huey, ARTFL‐LEFFTDS (ALLFTD), Megan S Barker

**Affiliations:** ^1^ Alpert Medical School of Brown University, Providence, RI, USA; ^2^ Butler Hospital, Providence, RI, USA; ^3^ Brown University, Providence, RI, USA; ^4^ Northwestern University Feinberg School of Medicine, Chicago, IL, USA; ^5^ Department of Psychiatry and Human Behavior, Alpert Medical School of Brown University, Providence, RI, USA; ^6^ ARTFL‐LEFFTDS Longitudinal Frontotemporal Lobar Degeneration (ALLFTD) Research Consortium, CA, USA

## Abstract

**Background:**

Behavioral variant frontotemporal dementia (bvFTD) is a neurodegenerative disorder marked by progressive changes in behavior, personality, and executive functioning. Predicting disease progression in bvFTD remains challenging due its heterogeneous nature. While age and genetics influence progression, the role of neuropsychiatric, motor, and cognitive symptoms is less understood. This study aims to identify symptoms most associated with fast disease progression in bvFTD over one year.

**Method:**

Data were collected from 192 individuals (mean age = 62.4, SD = 8.4, 37% female) with bvFTD from the ARTFL‐LEFFTDS Longitudinal Frontotemporal Lobar Degeneration study. Participants were classified into “fast” (*n* = 71) and “slow” (*n* = 121) progressors based on a >4 point increase in the FTLD‐modified Clinical Dementia Rating Sum of Boxes. Random forest modeling identified the top 7, 5, and 3 most important neuropsychiatric, motor, and cognitive features (out of 43) linked to fast disease progression. Model performances were compared using area under the curve (AUC), sensitivity (Se), specificity (Sp), positive predictive value (PPV), negative predictive value (NPV), and out‐of‐bag error rate (OOB). A logistic regression evaluated the incremental value of adding the identified features in addition to known demographic (e.g., age) and clinical variables (e.g., symptom duration).

**Result:**

The top 7 features associated with fast progression included impaired verbal memory (encoding then delayed recall), impaired working memory, compulsive behaviors, irritability, impaired letter fluency, and impaired mental flexibility. The top 5 features yielded the best model indices (AUC=0.75, Se=0.88, Sp=0.46, PPV=0.70, NPV=0.73, and OOB=26%) compared to the top 3 and 7. Logistic regression revealed that adding these 5 features significantly improved fast progression prediction above demographics and clinical traits (∆R2 = 0.12, p < .001).

**Conclusion:**

Impaired memory, impaired working memory, compulsive behaviors, and irritability were the strongest features of faster progression, and these features more effectively distinguished fast progressors from slow progressors than demographic and clinical variables alone. These findings could help guide early intervention strategies and enhance disease management, by highlighting key symptoms to target, supporting family education, and identifying fast progressors who may benefit from more urgent interventions.

